# Editorial: The Regulating Mechanisms of Development, Growth, and Metabolism: From Ground to Space

**DOI:** 10.3389/fcell.2022.951741

**Published:** 2022-06-15

**Authors:** Xiaohua Lei, Wensheng Zhang, Yan Zhang, Lei Zhao

**Affiliations:** ^1^ Center for Energy Metabolism and Reproduction, Shenzhen Institutes of Advanced Technology, Chinese Academy of Sciences, Shenzhen, China; ^2^ Cam-Su Genomic Resource Center, Medical College of Soochow University, Suzhou, China; ^3^ State Key Laboratory of Agrobiotechnology, College of Biological Sciences, China Agricultural University, Beijing, China; ^4^ Institute of Environmental Systems Biology, Dalian Maritime University, Dalian, China

**Keywords:** microgravity, cardiac remodelling, cell growth, embryonic development, *Arabidopsis thaliana*

It is an ambitious goal for human beings to realize the long-term survival of life in outer space, which has also become a Frontiers Research Topic of concern for the scientific community (Lei et al., 2020). During spaceflight, living organisms are inevitably exposed to a prolonged state of microgravity, which can trigger a series of responses to the growth and development of life at various levels from the molecular to the cellular and the whole organism (Pietsch et al., 2017; Prasad et al., 2020). Despite some advances in understanding the growth and development of mammals and plants in space, very few studies have focused on the cellular and molecular levels that mediate cell growth, development, and metabolism under microgravity conditions. This Research Topic manuscript investigates the impact of the space microgravity environment, in particular ground-based simulation-platform microgravity, on the development, cell proliferation and differentiation of living organisms at a cellular and individual level. To investigate this Research Topic, we will review six articles covering contents related to space life sciences including stem cells, cancer cells, and plant/animal growth in space or simulated microgravity (SMG). These studies highlight the molecular mechanisms with which these organisms respond and adapt themselves to the microgravity environment.

Microgravity affects prominently cardiovascular health, which is a gravity-dependence physical factor. In this Research Topic, two papers reveal the mechanism of microgravity-induced cardiac remodelling. It is well known that protein ubiquitination is an important multifaceted post-translational modification involved in cardiac remodelling (Zhang et al., 2020). Zhong et al. explored the role of WW domain-containing E3 ubiquitin protein ligase 1 (WWP1) in SMG-induced cardiac remodelling and in function decline in mice and monkeys. The authors found that WWP1-DVL2-CaMKII-HDAC4 pathway was activated in the heart of mice subjected to SMG and that WWP1 KO can protect mice from cardiac remodelling induced by SMG via inhibiting the DVL2-CaMKII-HDAC4 axis. The 3′untranslated region (3′-UTR) of casein kinase two interacting protein-1 (CKIP-1) is a pivotal mediator in pressure overload, which can induce cardiac remodelling (Zhao et al., 2021). Zhao et al. found that CKIP-1 3′-UTR remarkably attenuated cardiac dysfunction and mass loss in SMG by inhibiting lipid accumulation and elevating fatty acid oxidation-related gene expression in the mice’s hearts.

Accumulating evidence suggests that microgravity induces many changes in the proliferation, differentiation and growth behaviour of stem or cancer cells (Lei et al., 2018; Grimm et al., 2020). Ma et al. investigated the effect of microgravity on the hematopoietic differentiation of human embryonic stem cells (hESCs). The authors found that SMG helps hESCs differentiate into hemogenic endothelium progenitors (HEPs) and hematopoietic stem/progenitor cells (HSPC). They also observed that SMG supports the formation of 3D hematopoietic clusters. Mechanistically, SMG upregulated the key hematopoiesis-related gene expression and enriched the key metabolic pathways, including angiogenesis, HEME metabolism, glycolysis, and oxidative phosphorylation. Daniela Grimm and colleagues reported a 3D growth of prostate cancer cells (PCC) under SMG conditions (Dietrichs et al.). This study aimed to detect the gene expression and cytokine secretion of PCC exposed to SMG. They found that there are two types of cell growth, i.e., 3D multicellular spheroids (MCS) and adherent monolayer (AD). Interestingly, the treatment of PC-3 cells under SMG resulted in early activation of the VEGF pathway, downregulation of EGFR1, and downregulation of PAM signalling. In addition, proinflammatory cytokines IL1B, IL6, and CXCL8 were markedly upregulated and closely correlated with the first phases of spheroid formation in PC-3 cultivated cells under SMG conditions (Dietrichs et al.). Embryonic stem cells (ESCs) are derived from the inner cell mass of a blastocyst, which has the capability to self-renew and differentiate into all cell types, and are a great model for studying cell growth and development responses to microgravity (Lei et al., 2018). Li et al. review recent progress on the effects of microgravity on ESCs and early embryonic development, and propose some hypotheses regarding the potential mechanisms. The authors also discuss the controversies in this field and provide a critical overview of current work and prospects for the key questions concerning reproduction and early embryonic development in a space environment.

Finally, Wang et al. examine the effects of photoperiodic signals on the spaceflight response of the Arabidopsis during the reproductive stage. On board China’s recoverable satellite SJ-10, they grew Arabidopsis under long-day (LD) and short-day (SD) conditions. They constructed a transgenic plant containing FLOWERING LOCUS T (FT) and a green fluorescent protein (GFP) reporter gene under the control of a heat shock-inducible promoter (HSP17.4) to investigate the role of the FT expression in Arabidopsis at the reproductive stage during spaceflight. Additionally, a genome-wide analysis of the mRNA expression changes derived from the leaves of Arabidopsis grown under LD and SD in space was performed, and the results were compared with their controls grown on the ground. They found out that the biological functions of the photoperiod-related responsive genes were altered during spaceflight, including protein synthesis and post-translation protein modulation, notably protein phosphorylation. This work also suggested that circadian clock genes could play an important role in FT-integrated daylength pathways for plant adaptation during spaceflight at the reproductive developmental stage.

In summary, this report has reviewed the diverse roles of microgravity in cardiac remodelling and the growth of cells and plants, and the discussed papers analyze the molecular mechanisms of organisms in response to a microgravity environment.
